# Enterohemorrhagic *Escherichia coli* Specific Enterohemolysin Induced IL-1β in Human Macrophages and EHEC-Induced IL-1β Required Activation of NLRP3 Inflammasome

**DOI:** 10.1371/journal.pone.0050288

**Published:** 2012-11-27

**Authors:** Xiaoai Zhang, Yuli Cheng, Yanwen Xiong, Changyun Ye, Han Zheng, Hui Sun, Hongqing Zhao, Zhihong Ren, Jianguo Xu

**Affiliations:** 1 National Institute for Communicable Disease Control and Prevention, Chinese Center for Disease Control and Prevention, State Key Laboratory for Infectious Disease Prevention and Control, Changping, Beijing, China; 2 Beijing Center for Disease Prevention and Control, Beijing, China; The University of Texas Health Science Center at San Antonio, United States of America

## Abstract

Enterohemorrhagic *Escherichia coli* (EHEC) O157:H7 is a major foodborne pathogen causing hemorrhagic colitis and hemolytic-uremic syndrome. The role of EHEC O157:H7-enterohemolysin (Ehx) in the pathogenesis of infections remains poorly defined. In this study, we used gene deletion and complement methods to confirm its putative functions. Results demonstrated that, in THP-1 cells, EHEC O157:H7-Ehx is associated with greater production of extracellular interleukin (IL)-1β than other cytokines. The data also showed that EHEC O157:H7-Ehx contributed to cytotoxicity in THP-1 cells, causing the release of lactate dehydrogenase (LDH). Although we observed a positive correlation between IL-1β production and cytotoxicity in THP-1 cells infected with different EHEC O157:H7 strains, our immunoblot results showed that the majority of IL-1β in the supernatant was mature IL-1β and not the pro-IL-1β that can be released after cell death. However, EHEC O157:H7-Ehx had no detectable effect on biologically inactive pro-IL-1β at the mRNA or protein synthesis levels. Neither did it affect the expression of apoptosis-associated speck-like protein containing a CARD (ASC), caspase-1, or NOD-like receptor family pyrin domain containing 3 (NLRP3). RNA interference experiments showed that EHEC O157:H7-induced IL-1β production required the involvement of ASC, caspase-1, and NLRP3 expression in THP-1 cells. Our results demonstrate that Ehx plays a crucial role in EHEC O157:H7-induced IL-1β production and its cytotoxicity to THP-1 cells. NLRP3 inflammasome activation is also involved in EHEC O157:H7-stimulated IL-1β release.

## Introduction

Enterohemorrhagic *Escherichia coli* (EHEC) serotype O157:H7 is a major foodborne pathogen. It causes diarrhea, hemorrhagic colitis, and hemolytic-uremic syndrome (HUS), which can be life-threatening [1]. Macrophages were previously shown to contribute to the cytokine production that is associated with HUS. In the large intestine, EHEC O157:H7 can come into contact with underlying human macrophages through the follicle-associated epithelium of Peyer’s patches [2]. When the intestinal epithelial cells are damaged, EHEC O157:H7 can penetrate the basement membrane and come into contact with macrophages. Previous studies have shown that tumor necrosis factor-α (TNF-α) and interleukin (IL)-1β produced by infected macrophages can contribute to the severe inflammation associated with HUS [3]. More studies focused on the better-known virulence factors of EHEC O157:H7 that contribute to the inflammatory response, such as Shiga toxins (Stxs), the locus of enterocyte effacement (LEE) pathogenicity island and flagellin [4]–[8]. However, the interactions between EHEC O157:H7 and human macrophages have not been well characterized. The role of virulence factors in the macrophage-associated inflammatory response to EHEC O157:H7 infection remains to be determined.

Almost all clinical isolates of EHEC O157:H7 possess a virulence plasmid called pO157 [1]. The sequence of pO157 contains 100 open reading frames (ORFs) [9]. Among them, some putative virulence genes have been characterized previously. These include an enterohemolysin (*ehx*), a catalase-peroxidase (*katP*), a type II secretion system apparatus (*etp*), a serine protease (*espP*), a putative adhesin (*toxB*), a zinc metalloprotease (*stcE*), and an *eae* conserved fragment (*ecf*) [10]–[16]. Genome-wide transposon mutagenesis revealed that *espP* and *ehxD* were directly involved in biofilm formation and were also important for adherence to T84 intestinal epithelial cells, suggesting a role for these genes in tissue interactions *in vivo* [17]. Antibodies against enterohemolysin (Ehx) have been detected in the sera of patients with HUS, suggesting that it is an important immunogenic protein and that it interacts with the host immune system [18]. In this study, we examined the immunogenic role of Ehx encoded on virulence plasmid pO157 of EHEC O157:H7 ELD933. Results showed that Ehx activated human macrophages and caused them to produce mature IL-1β. EHEC O157:H7-induced release of IL-1β required the involvement of apoptosis-associated speck-like protein containing a CARD (ASC), caspase-1, and NOD-like receptor family pyrin domain containing 3 (NLRP3).

**Table 1:** Primers.

Primer	Forward (5′-3′)	Reverse (5′-3′)
oriR	TTCTGAGGCAGGCTGGTATT	TGTTGCTTGTGCGGTATTGT
repB	ACAATACCGCACAAGCAACATGTCAGGCAGATGGAAAGCT	GACCACGATCACATAAGCAG
*ehxA*-1,2	ATGACAGTAAATAAAATAAAGAACATTTTCAATAATGCGAATGAGCCATATTCAACGGGA	ATTTCCAACTCTCTTAAATGCGATATCATCAAAGCTAATATTAGAAAAACTCATCGAGCA
*ehxA*-3,4	TTCAGGCAATACCATCAT	CAACGCAGGTAAAGAAAT
*ehxA*-5,6	ACGCACATACAGGAACAA	CTAACTCCCGCAGATACA
*ehxA*hb	CATGGTACCGACAGTAAATAAAATAAAGAAC ATT	GACTCTAGATCAATGATGATGGTGATGGTGGACAGTTGTCGTTAAAGTTGTTG
GAPDH [24]	GGTATCGTGGAAGGACTCATGAC	ATGCCAGTGAGCTTCCCGTTCAGC
IL-1β	GGCGGCATCCAGCTACGAATCTC	GGCGGCATCCAGCTACGAATCTC
casp-1	AGTTTGAAGGACAAACCGAAGGT	AGTTTGAAGGACAAACCGAAGGT
ASC	TGGATGCTCTGTACGGGAAGGTC	TCAGGATGATTTGGTGGGATTGC
NLRP3	ATCAGTATTGAGCACCAGCCATT	AGAGTGTTGCCTCGCAGGTAAAG
AIM2	GGCACAGTGGTTTCTTAGAGGTA	GCTGAGTTTGAAGCGTGTTGATC
NOD2	CTCTGTGCGGACTCTACTCTTTG	GTCACCACCTTGCGGGCATTCTT
NLRC3	ACCAACATCATCCGTGGCAACCT	TCGGGGAACATCTGCTCCAAACA
NLRC4	GAAGGAGACTTGGACGATTTGGC	CAGGACAGGTTCTGTGAAGGTGC
NLRC5	CCTATCAACTGCCCTTCCACAAT	CTCTATCTGCCCACAGCCTACCA

## Materials and Methods

### Bacterial Strains and Plasmids

The EHEC O157:H7 reference strain used in this study was EDL933 (ATCC 43895) (here called WT) [19]. Plamids pMD20-T, haboring an ampicillin (Amp) resistant gene, pUCK-T, harboring a kanamycin (Km) resistant gene and promotor pBAD24 induced by arabinose, were used as vectors. Plasmid pKOBEG is a thermosensitive replicon that carries the λ phage red γβα operon expressed under the control of the pBAD promoter [20]. The bacteria were grown in Luria-Bertani (LB) broth or on LB plates (pH 7.4). Chloramphenicol (50 µg/ml), Km (50 µg/ml), Amp (100 µg/ml), L-arabinose (10 mM) were added as needed.

**Figure 1:**
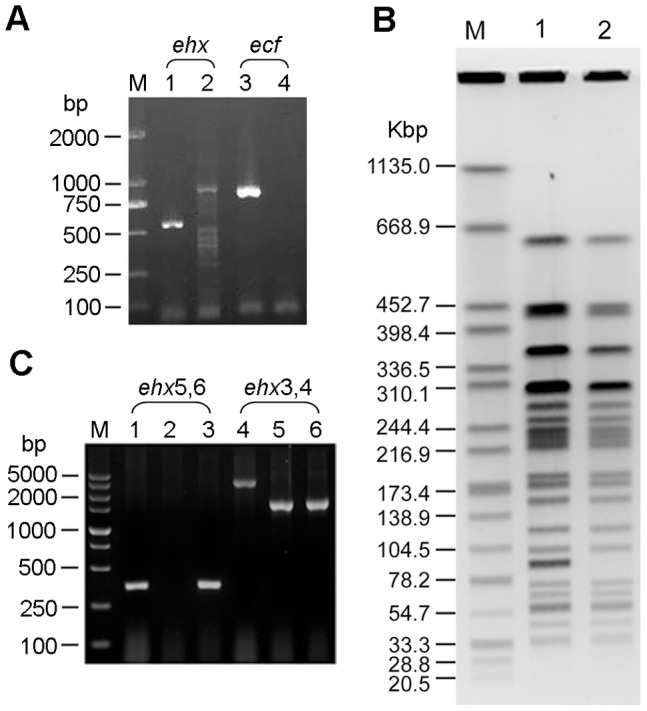
Construction of the mutant strains. (A) The genes *ehx* and *ecf* were detected in EDL933 but not in ΔpO157. Lane M: marker; Lanes 1 and 3: EDL933; Lanes 2 and 4: ΔpO157 mutant. (B) Comparison of genomic DNA of EDL933 and ΔpO157 using PFGE of *Xba*I-digested. Lane M: marker; Lane 1: EDL933; Lane 2: ΔpO157 mutant. (C) Using primer *ehxA*-3,4, EDL933 was amplified as a ≈3.3 kb fragment. Δ*ehxA* and Δ*ehxA*/pehxA were amplified as a reduced fragment to ≈1.6 kb. Using primer *ehxA-*5,6, Δ*ehxA* showed no PCR product. EDL933 and Δ*ehxA*/pehxA were amplified as a ≈360 bp fragment. Lane 1: EDL933; Lane 2: Δ*ehxA*; Lane 3: Δ*ehxA*/pehxA.

### Elimination of Virulence Plasmid pO157

The virulent 92-kb plasmid pO157 was eliminated from EDL933 using plasmid incompatibility. The resulting plasmid-free strain is here called ΔpO157. Briefly, two putative replication origins, *oriR* and *repB*, were amplified from purified EDL933 template by PCR using primers oriR and repB (Table 1) [9]. The PCR products of *oriR* and *repB* were cloned into pMD20-T vector and pUCK-T vector, respectively. pMD20-oriR and pUCK-repB were introduced into wild-type EDL933 by transformation. Transformants were isolated on LB agar containing Amp and Km and selected for loss of pO157 using agarose gel electrophoresis analysis. Amp-resistant and Km-resistant transformants were cured of pMD20-oriR and pUCK-repB by subculturing in LB broth without Amp and Km. The absence of pO157 was confirmed by PCR with primers for the pO157-specific genes *ecf* and *ehx* [21], [22]. The integrity of chromosomal DNA was confirmed using Pulsed field gel electrophoresis (PFGE).

**Figure 2:**
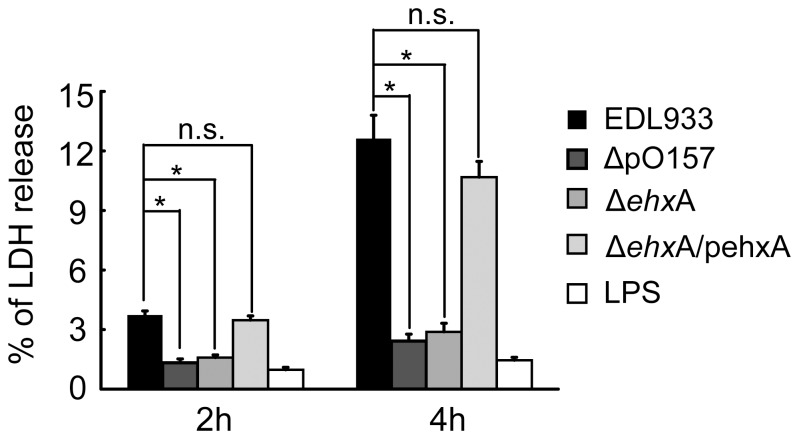
Cytotoxicity of human macrophages as indicated by the release of lactate dehydrogenase (LDH). Differentiated THP-1 cells were exposed to different bacterial strains (EDL933, ΔpO157, Δ*ehxA*, Δ*ehxA*/pehxA) for 2 and 4 h. The release of LDH was assessed at specific times during incubation. Data are shown as mean ± S.D. of experiments performed in triplicate. Significant differences (* *P<*0.05) are indicated. n.s., no significant differences (*P*>0.05).

**Figure 3:**
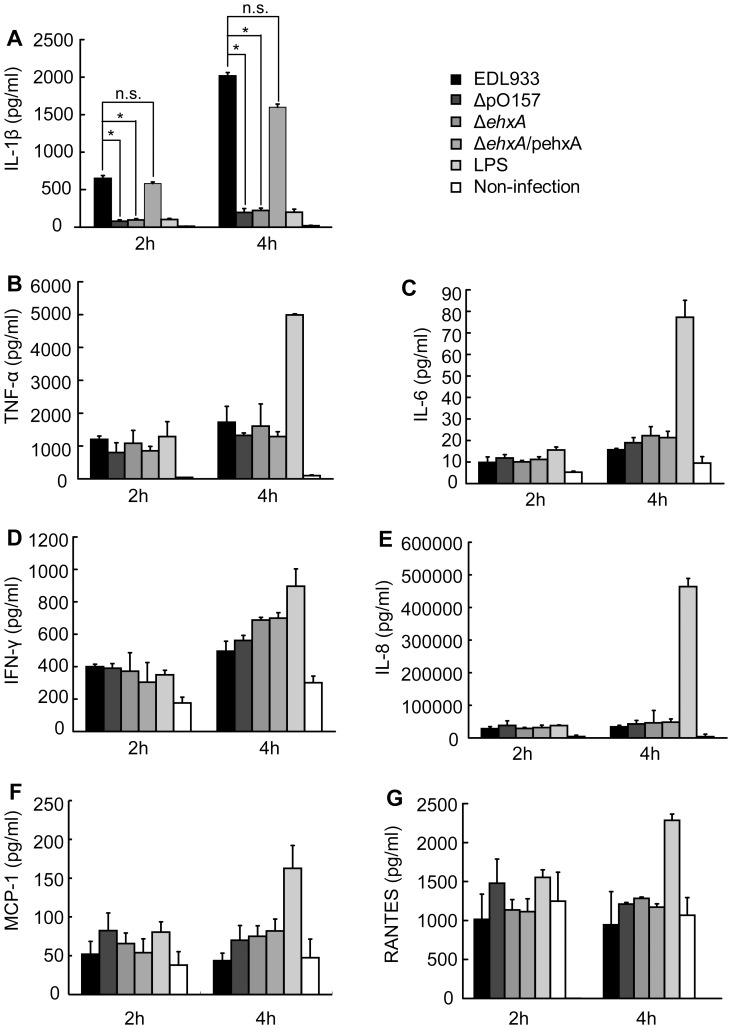
Effects of EHEC O157:H7 enterohemolysin on the production of IL-1β. Differentiated THP-1 cells were infected with EDL933, ΔpO157, Δ*ehxA*, Δ*ehxA*/pehxA, and LPS for 2 or 4 h. Concentrations of interleukin (IL)-1β, IL-6, IL-8, chemokine CC motif ligand 5 (RANETS/CCL5), monocyte chemotactic protein-1 (MCP-1), tumor necrosis factor-α (TNF-α), and Interferon-gamma (IFN-γ) were measured using ELISA. Values are expressed as mean ± S.D. of triplicate experiments. Significant differences (* *P*<0.05) were indicated. n.s., no significant differences (*P*>0.05).

### Construction of the *ehxA* Gene Deletion Mutant

The EDL933 *ehxA* deletion mutant (Δ*ehxA*) was constructed using the linear recombination (λRed) method described by Datsenko and Wanner [20]. Briefly, the primer *ehxA*-1,2 (Table 1) was used to amplify the Km resistance cassette from plasmid template pRS551 using PCR. The resulting product was then transformed by electroporation into EDL933-competent cells. EDL933 carrying pKOBEG, grown at 30°C in the presence of 10 mM arabinose. pKOBEG was removed by shaking for 15 minutes in a water bath at 42°C. Mutants were selected on LB-Km plates and confirmed by PCR using primers *ehxA*-3,4 and *ehxA*-5,6 (Table 1). A strain with the *ehxA* gene complement was created using arabinose-inducible expression vector pBAD24. *The ehxA* gene was amplified from purified EDL933 template using PCR with primer *ehxA*hb (Table 1). The gene was inserted into the *Xba*I-*Kpn*I sites of pBAD24 and transformed by electroporation into the donor strain Δ*ehxA*, creating a strain called Δ*ehxA*/pehxA. The complementary nature of the strain was confirmed by PCR.

**Figure 4:**
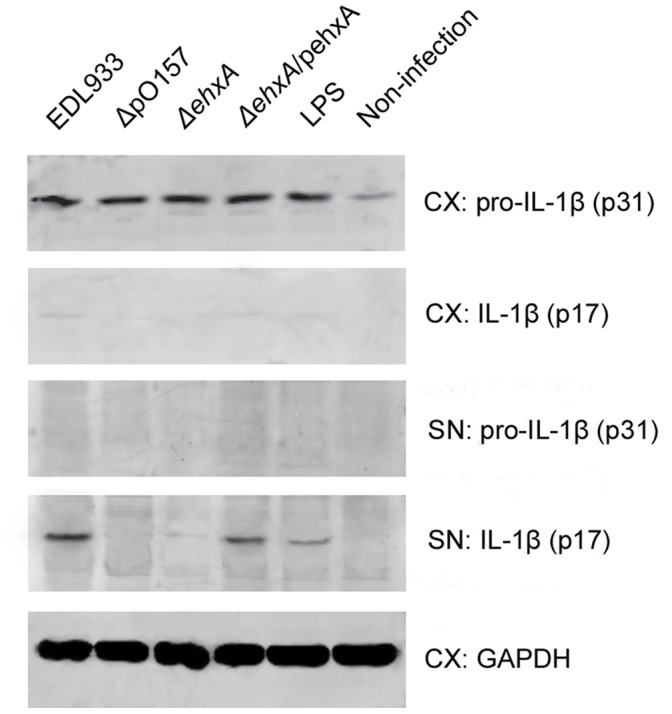
Pro-IL-1β and mature IL-1β in cell extract and supernatant as visualized by Western blotting. At 4 h after infection, pro-IL-1β and IL-1β in cell extracts (CX) and supernatants (SN) were visualized by Western blot analysis.

### Cell Culture and Infection

The human monocytic cell line THP-1 (ATCC TIB-202) was maintained and infected as described previously [23]. A total of 5×10^5^ cells were seeded in a 24-well plate and they differentiated into macrophage-like THP-1 cells after addition of 10^−7^ M phorbol 12-myristate 13-acetate (PMA) (Sigma-Aldrich, Louis, MO, U.S.) for 48 h of culture. The differentiated THP-1 cells were cultured in fresh medium (RPMI 1640, Invitrogen, Carlsbad, CA, U.S.) containing 10% fetal bovine serum (Invitrogen) and washed three times with medium before infection. The bacteria were prepared by shaking overnight at 37°C in LB broth. Concentrations of bacteria were determined by measuring absorbance at an optical density 600 nm. The bacterial cells were washed three times and then diluted in reduced serum medium (GIBCO, Carlsbad, CA, U.S.). Aliquots of bacteria were added in triplicate to the cell monolayer at a multiplicity of infection (MOI) of 10 and then incubated at 37°C in a 5% CO_2_ atmosphere.

**Figure 5:**
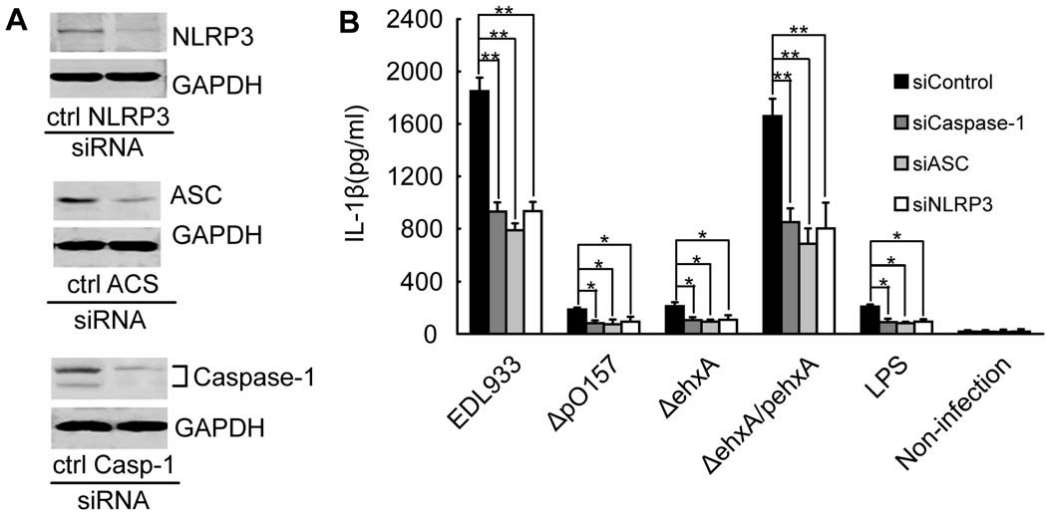
Roles of caspase-1, apoptosis-associated speck-like protein containing a CARD (ASC), and the NOD-like receptor family pyrin domain containing 3 (NLRP3) in EHEC O157:H7-induced IL-1β production. THP-1 cells were transfected with control siRNA or siRNA specific to caspase-1, ASC, or NLRP3, respectively. After 48 h, cells were infected with EDL933, Δ*ehxA*, ΔpO157, and Δ*ehxA*/pehxA, respectively. (A) Knockdown of caspase-1, ASC, and NLRP3, was assayed by Western blotting. (B) Cell culture supernatants were collected 4 h after infection and subjected to IL-1β ELISA. Results represent the mean ± S.D. of three independent experiments. Significant differences (**p<0.01, *P<0.05) were indicated. n.s., no significant differences (*P*>0.05).

### Cytotoxicity Assay

At 2 and 4 h postinfection, the supernatant was collected and the release of lactate dehydrogenase (LDH) was quantified using a Cytotox96 Kit according to the manufacturer’s instructions (Promega, Madison, WI, U.S.). The relative level of cytotoxicity was expressed as (experimental release – spontaneous release)/(maximum release – spontaneous release) × 100%. Spontaneous release was here defined as the amount of LDH activity in the supernatant of uninfected cells and the maximum release was here defined as the amount of LDH activity when cells were lysed with lysis buffer.

**Figure 6:**
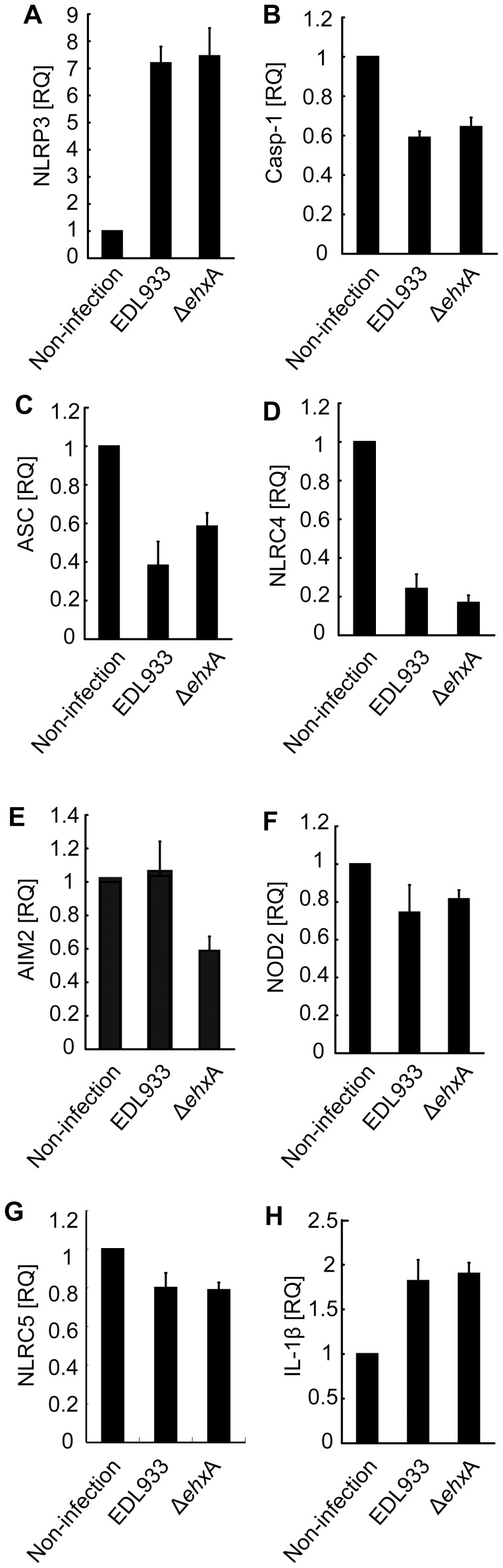
Expression of inflammasome components in differentiated THP-1 cells. Differentiated THP-1 cells were left untreated or were infected with EDL933 or Δ*ehxA*. They were then lysed over 4 h postinfection. mRNA expression of selected genes was analyzed using RT-PCR.

**Figure 7:**
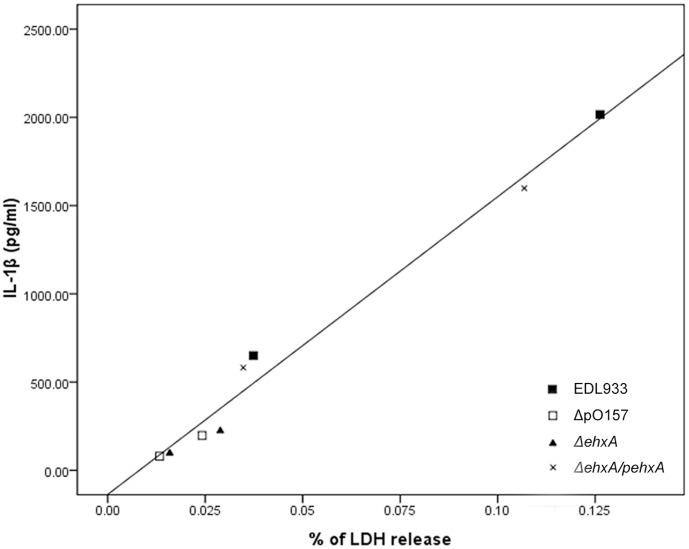
Correlation between the release of LDH and concentration of IL-1β in THP-1 cells infected with EHEC O157:H7. A significant positive correlation was observed (*P*<0.01).

### Cytokine Assay

At 2 and 4 h postinfection, the supernatants of the cell cultures were collected and the levels of human cytokines IL-6, IL-8, chemokine CC motif ligand 5 (RANETS/CCL5), monocyte chemotactic protein-1 (MCP-1), TNF-α, interferon-gamma (IFN-γ), and IL-1β were quantified using a Luminex Kit in accordance with the manufacturer’s instructions (R&D Systems, Minneapolis, MN, U.S.). Differentiated THP-1 with culture medium alone served as a control for the spontaneous release of cytokine. LPS (1 µg/ml) (*E. coli* O111, Sigma) served as a positive control.

### RT-PCR Analysis

At 2 and 4 h postinfection, total RNA from differentiated THP-1 cells was isolated using an RNeasy Mini Kit (Qiagen, Hilden, Germany) and digested with RNase-free DNase I (Promega). Then cDNA was synthesized using Superscript II reverse transcriptase and random hexamers according to the manufacturer’s guidelines (TaKaRa Bio, Dalian, China). The cDNA was amplified using semiquantitative PCR using SYBR green I master mix (TakaRa) and specific primers (Table 1) using the Rotor-Gene Q (Qiagen). Relative expression of target genes were calculated as 2^−ΔΔ*CT*^. ΔΔ*CT* = [(*CT* gene of interest – *CT* internal control) sampleA – (*CT* gene of interest – *CT* internal control) sampleB] [24]. The mRNA expression level of each target gene was normalized to that of glyceraldehyde-3-phosphate dehydrogenase (GAPDH).

### RNA Interference

siNLRP3, siASC, siCaspase-1, and siControl were synthesized as previously published [25]. A total of 5×10^5^ THP-1 cells were differentiated with PMA in a 12-well plate. Then the cells were transfected using the RNAiMAX with 48 pmol siRNAs per well according to the manufacturer’s protocol (Invitrogen). After 48 h of incubation at 37°C (5% CO_2_ atmosphere), the cells were further infected with bacteria at a MOI of 10 in 1.8 ml complete RPMI 1640.

### Western Blot Analysis

Cell-free supernatants were concentrated using Amicon Ultra-4 10K Centrifugal Filter Devices (Millipore, Bedford, MA, U.S.). Cell extracts and concentrated supernatants were separated using SDS-PAGE and blotted. Membranes were first exposed to antibodies specific to IL-1β (no. sc-52012; Santa Cruz, CA, U.S.), caspase-1 (no. sc-56036), apoptosis-associated speck-like protein containing a CARD (ASC) (no. sc-271054), NOD-like receptor family pyrin domain containing 3 (NLRP3) (no. ALX-804-881; Enzo, UK), or GAPDH (no. sc-137179; Santa Cruz, CA, U.S.). They were incubated with secondary antibodies (IRDye 800-labeled anti-mouse IgG or anti-rabbit IgG; no. 610-132-121 or 611-132-002; Rockland, Gilbertsville, PA, U.S.). Proteins were detected using an Odyssey Infrared Imaging System (LI-COR, Lincoln, NE, U.S.).

### Statistical Analysis

Statistical analysis was performed using one-way ANOVA with Newman–Keuls post-testing. The correlation between LDH level and concentration of IL-1β in supernatants of THP-1 cells infected with EHEC O157:H7 was assessed using Pearson’s test and linear regression. Values of *P*≤0.05 were considered significant.

## Results

### Construction of EHEC O157:H7 Enterohemolysin Gene *ehxA* Deletion Mutant

The virulent plasmid eliminated derivative strain of EHEC O157:H7 EDL933, ΔpO157, was confirmed by PCR. *ehx* and *ecf* were detected in EDL933 but not in ΔpO157 (Figure 1A). PFGE analysis of chromosomal DNA after digestion with *Xba*I showed that the ΔpO157 mutant strain differed from the EDL933 by the absence of a 92 kb band (Figure 1B). The *ehxA* gene deletion mutant of EDL933, Δ*ehxA*, was constructed by replacing the *ehxA* genes on the plasmid with a Km resistance gene. Using primer *ehxA*-3,4, the *ehxA* region of the EDL933 was amplified as a ≈3.3 kb fragment. The Δ*ehxA* region of the complement strain Δ*ehxA*/pehxA caused a reduction of the size of the corresponding PCR product to ≈1.6 kb (Figure 1C). When primer *ehxA-*5,6, an internal segment of gene *ehxA*, was used, Δ*ehxA* produced no PCR product and the complement strain Δ*ehxA*/pehxA was amplified as a ≈360 bp fragment (Figure 1C).

### Association of EHEC O157:H7-Ehx and the Release of LDH from Human THP-1 Cells

The THP-1 cells were infected with EDL933, its virulent plasmid-elimination derivative strain (ΔpO157), its *ehxA* deletion mutant (Δ*ehxA*), and its *ehxA* complement strain (Δ*ehxA*/pehxA). Then the release of LDH was examined 2 and 4 h post-infection. There was a significant difference between the release of LDH from EDL933 and from ΔpO157 and between Δ*ehxA* and Δ*ehxA*/pehxA (*P<*0.05) (Figure 2). These results showed that the EhxA encoded on pO157 was toxic to THP-1 cells and involved in the release of LDH from THP-1 cells.

### EHEC O157:H7-Ehx Induced IL-1β Release in THP-1 Cells

The IL-1β production in the supernatants of cell culture and cell extract infected with different bacterial strains (EDL933, ΔpO157, Δ*ehxA*, Δ*ehxA*/pehxA) was tested by ELISA and Western-blot. The ELISA results showed that the THP-1 cells stimulated by EDL933 produced higher level of IL-1β in supernatant compared with the cells stimulated by its virulence plasmid elimination derivative strain ΔpO157 (*P*<0.05), and its *ehxA* gene deletion mutant Δ*ehxA* (*P*<0.05). The reduced release of IL-1β from the *ehxA* gene deletion mutant can be restored when complemented with *ehxA* gene (Δ*ehxA*/pehxA) (*P*<0.05) (Figure 3A). We also assessed production of IL-6, IL-8, RANETS/CCL5, MCP-1, TNF-α, and IFN-γ in THP-1 cells stimulated by those strains, and results showed that EhxA had no effect on production of the other cytokines we examined (Figure 3B–G).

To confirm whether the presence of IL-1β production we analyzed in the supernatant using ELISA was the biologically active mature form or the biologically inactive pro-IL-1β, which can be released passively during cell lysis due to cytotoxicity, we examined the production of pro-IL-1β and mature-form IL-1β in both supernatants and cell lysis using immunoblotting after the cells had been infected with different strains (EDL933, ΔpO157, Δ*ehxA*, and Δ*ehxA*/pehxA). Results showed that the IL-1β in the supernatant was mainly mature-form p17 and the IL-1β in the cell lysis was mainly inactive-form pro-IL1β, as shown in Figure 4. We also observed that the THP-1 cells stimulated by EDL933 showed significantly higher levels of mature-form IL-1β (p17) in the supernatant than cells stimulated by its virulence plasmid elimination derivative strain ΔpO157 or its *ehxA* gene deletion mutant Δ*ehxA* (Figure 4). The reduced release of mature IL-1β (p17) from the *ehxA* gene deletion mutant was restored when complemented with *ehxA* gene (Δ*ehxA*/pehxA) (Figure 4). However, neither the expression of intracellular IL-1β mRNA (Figure S1) nor pro-IL-1β protein in cell lysis differed across the four strains (Figure 4). Overall, these results suggest that EhxA encoding on pO157 was responsible for the higher levels of extracellular production of mature IL-1β in THP-1 cells but had no effect on intracellular production of biologically inactive pro-IL-1β in THP-1 cells.

### Role of ASC, NLRP3, and Caspase-1 in EHEC O157:H7-induced IL-1β Production

The involvement of the inflammasome components ASC, NLRP3, and caspase-1 in the EHEC O157:H7-induced release of IL-1β was assessed using siRNA and immunoblotting. The results showed that the levels of IL-1β in supernatants in cells treated with ASC, caspase-1, or NLRP3 siRNA were all significantly lower than those of cells treated with control siRNA infected with EDL933, ΔpO157, Δ*ehxA*, and Δ*ehxA*/pehxA (Figure 5A, 5B). This suggests that ASC, NLRP3, and caspase-1 are required for the EHEC O157:H7-induced release of IL-1β but the evidence is not sufficient to conclude that EHEC O157:H7-induced IL-1β production takes place in a ASC-, NLRP3-, or caspase-1-dependent manner in this siRNA system.

### Expression of Inflammasome Components in EHEC O157:H7-infected THP-1 Cells

To explore if EHEC O157:H7 activates one or more inflammasomes, we assessed the expression of several inflammasome components in EHEC O157:H7-infected THP-1 cells by RT-PCR using specific primers. The results showed that all target genes were expressed in THP-1 cells infected with different strains. However, in EHEC O157:H7-infected THP-1, only the NLRP3 and IL-1β transcripts were found to be upregulated. However, EhxA had no effect on the mRNA expression of any inflammasome component in THP-1 cells infected with EDL933 (Figure 6).

### Correlation between EhxA-induced Cytotoxicity and IL-1β Secretion by THP-1 Cells

Although we have ruled out the possibility that cytotoxicity of EHEC O157:H7 is the main cause of the increase in the release of IL-1β into the supernatant, we still noticed a significant positive correlation between IL-1β production and the release of LDH in the supernatants of THP-1 cells infected with different strains (r = 0.991, *P*<0.01) (Figure 7). This suggests that cytotoxicity of EhxA might contribute to some extent to the higher levels of extracellular IL-1β production in supernatant from EHEC O157:H7-infected THP-1 cells but that the effect of EhxA on processing the pro-IL-1β to mature IL-1β is still the main mechanism by which mature Il-1β is released.

## Discussion

Although there is a growing body of evidence regarding the virulence factors of EHEC O157:H7, such as Stxs and flagellin in epithelial cells, the role of specific Ehx encoding on plasmid of EHEC O157:H7 in pathogenesis has not been fully elucidated. It is likely that the EHEC-Ehx is expressed during human infection and subsequent disease, as patients suffering from O157-associated HUS produce specific EHEC-Ehx antibodies in almost all cases [18].

The EHEC-Ehx is a highly active repeats-in-toxin with pore-forming capacity similar but not identical to that of chromosomal encoded *E. coli* α-hemolysin. The presence of α-hemolysin in enteroaggregative and cytodetaching *Escherichia coli* strains appears to play a critical role in both oncosis in human monocyte-derived macrophages and apoptosis in the murine macrophage cell line (J774 cells) [26]. The hemolysin A of *E. coli* was found to increase the permeability of human macrophages by forming ionic pores [27]. Bauer and Welch found that EHEC-Ehx lysed bovine but not human lymphoma cells. They hypothesized that the target cell specificity of EHEC-Ehx might be narrow [28]. Kartch’s group has reported that the EHEC-Ehx is cytotoxic to human brain microvascular endothelial cells and that this toxicity may contribute to the virulence of the *stx*-negative *E. coli* O26 strains [29]. Our data provide clear evidence that EHEC-Ehx encoded on the plasmid of EDL933 contributed to the cytotoxicity of EHEC in THP-1 cells. Macrophages are the main producers of proinflammatory cytokines in response to bacterial infection and the cytotoxicity of the macrophages can affect the host immune response to bacterial invasion and affect the pathogenesis of EHEC O157:H7 infection.

Previous studies have shown that the inflammatory response is involved in the pathogenesis of EHEC O157:H7 infection [30]–[32]. HUS patients show an increase in a variety of circulating proinflammatory cytokines, such as IL-1β, TNF-α, and IL-8, in response to EHEC O157:H7 infection [30]–[32]. However, which components of EHEC O157:H7 contribute to the elevated level of specific pro-inflammatory cytokines through macrophage activity has not been well demonstrated. In this study, we demonstrated that the EHEC-Ehx induced a higher level of mature IL-1β in THP-1 cells. Other cytokines (IL-6, IL-8, RANETS/CCL5, MCP-1, TNF-α, and IFN-γ) were also examined and none of them were induced by Ehx.

IL-1β is an important proinflammatory mediator. It exerts a variety of biological effects. During EHEC O157:H7 infection, IL-1β is a potent inducer of fever and inflammatory response. It can disrupt the intestinal barrier, permitting transport of Stxs into the circulatory system [33]. IL-1β was also found to be involved in HUS through increasing expression of Gb3, the receptor of Stx on endothelial cells allowing increased binding of Stx [3], [34]. In this study, we observed that EHEC-Ehx could contribute to the release of mature IL-1β by THP-1 cells.

To determine the mechanism underlying the EHEC O157:H7-Ehx-induced release of IL-1β, we investigated how Ehx might play a role in each step of the release of IL-1β. The mechanism underlying the release of IL-1β has three major steps: 1) Synthesis the biologically inactive pro-IL-1β. 2) Cleavage of pro-IL-1β by caspase-1 processing into mature biologically active IL-1β. 3) Secretion of mature IL-1β into extracellular milieu [35]. First, we found that Ehx had no effect on intracellular gene expression and production of biologically inactive pro-IL-1β in THP-1 cells by RT-PCR and immunoblotting. These data imply that EhxA may affect the subsequent steps in the release of IL-1β release. Second, we demonstrated that the NLRP3/ASC/caspase-1 inflammasome is required for EHEC O157:H7-induced IL-1β production using RNA interference experiments. The cysteine protease caspase-1 is responsible for the proteolytic processing and secretion of IL-1β. The inflammasome is a multi-protein complex critical to the activation of caspase-1 and induction of inflammatory responses. The inflammasome complex includes at least one NLR and an adaptor protein called ASC, which links the NLR to procaspase-1. The NLRP3 inflammasome has been reported to be activated by bacterial pore-forming toxins [36]–[40]. In this study, although our current data demonstrated that EHEC O157:H7-induced Il-1β was only partially dependent on caspase-1/ASC/NLRP3 inflammasome, the evidence was not sufficient to support the conclusion that EHEC O157:H7 could induce the release of IL-1β through any caspase-1-dependent or -independent pathway. This is because neither caspase-1 nor ASC nor NLRP3 was completely silenced in these assays. Further experiments using gene knock-out mice are necessary to determine the role of these inflammasomes in EHEC-induced IL-1β. Third, different exocytosis pathways have been observed in monocytes, macrophages, and dendritic cells. These pathways export the cytokine IL-1β, one of which is the type of IL-1β released upon cell lysis [41]. In this study, we found a positive correlation between IL-1β production and cytotoxicity induced by EHEC-Ehx. Even the cytotoxicity of Ehx has been found to contribute to the release of IL-1β through cell lysis, which cannot be the main source of extracellular IL-1β because most of the IL-1β in the supernatant was biologically active mature IL-1β, as shown by immunoblot analysis. Further experiments are needed to determine the mechanism by which cytotoxicity of Ehx affects the secretion of mature IL-1β into the extracellular space and how cytotoxic Ehx affects the pathogenesis of EHEC infection.

In this study, we found EHEC O157:H7-Ehx to contribute to cytotoxicity in THP-1 cells. It was also found responsible for higher levels of mature IL-1β. The NLRP3 inflammasome was found to mediate EHEC O157:H7-activated IL-1β production. Ehx may activate pro-caspase-1 through activation of NLRP3, like other pore-form bacteria toxins. However, the possibility that other types of inflammasome signaling may be activated by Ehx cannot yet be ruled out. This may also have stimulated the release of IL-1β. Cytotoxicity to THP-1 cells may also contribute to the release of IL-1β using some as yet unknown mechanism. Further study is needed to determine the possible roles of IL-1β in the pathogenesis of this potentially fatal foodborne infection.

## Supporting Information

Figure S1 **mRNA expression of IL-1β in differentiated THP-1 cells.** Differentiated THP-1 cells were left untreated or were infected with EDL933, ΔpO157, Δ*ehxA*, or Δ*ehxA*/pehxA. Cells were lysed over 2 h or 4 h postinfection mRNA expression of IL-1β was analyzed using RT-PCR.
(TIF)
