# Supplementary figures and images for: Enterohemorrhagic Escherichia coli Specific Enterohemolysin Induced IL-1β in Human Macrophages and EHEC-Induced IL-1β Required Activation of NLRP3 Inflammasome
Source: PLoS One. 2012 Nov 27;7(11):e50288. doi: 10.1371/journal.pone.0050288 (PMC3507778; doi:10.1371/journal.pone.0050288)

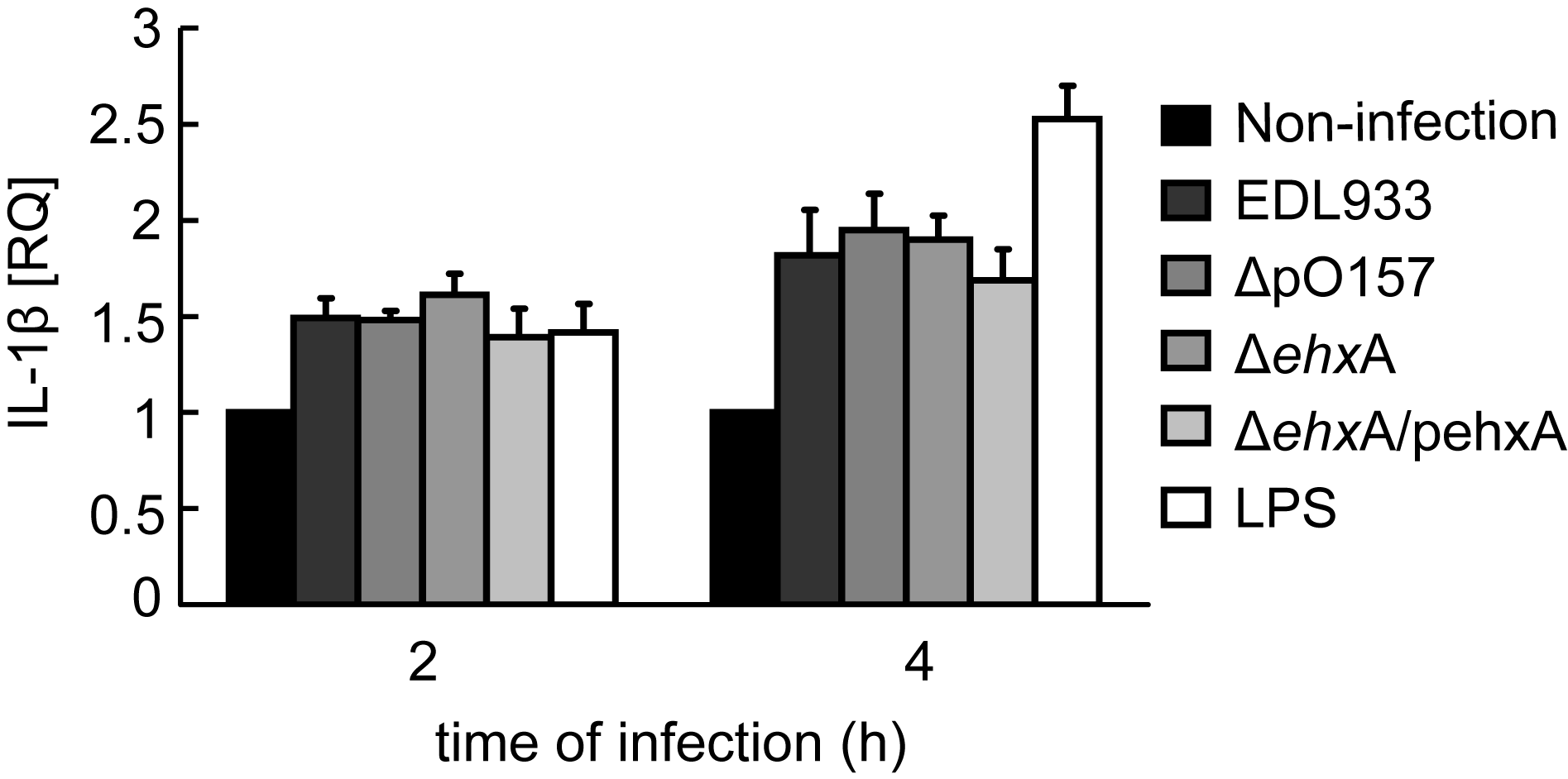

Supplement: Figure S1 — mRNA expression of IL-1β in differentiated THP-1 cells. Differentiated THP-1 cells were left untreated or were infected with EDL933, ΔpO157, ΔehxA, or ΔehxA/pehxA. Cells were lysed over 2 h or 4 h postinfection mRNA expression of IL-1β was analyzed using RT-PCR. (TIF) [file pone.0050288.s001.tif]
